# Neuroticism is positively associated with leptin/adiponectin ratio, leptin and IL-6 in young adults

**DOI:** 10.1038/s41598-021-89251-y

**Published:** 2021-05-07

**Authors:** Mikaela Syk, Johan Isaksson, Annica J. Rasmusson, Lisa Ekselius, Janet L. Cunningham

**Affiliations:** 1 Department of Neuroscience, Psychiatry, Akademiska sjukhuset, Uppsala University, 751 85 Uppsala, Sweden; 2 Department of Neuroscience, Child and Adolescent Psychiatry, Uppsala University, Uppsala, Sweden

**Keywords:** Emotion, Psychology, Endocrinology, Medical research, Risk factors, Cytokines, Inflammation

## Abstract

High neuroticism is related to cardiovascular morbidity. Early detection of metabolic and cardiovascular risk is important in high-risk groups to enable preventive measures. The aim of this study was therefore to explore if neuroticism is associated with early biomarkers for cardiovascular and metabolic disease in young adults from a psychiatry cohort. Blood samples and self-ratings on neuroticism with the Swedish universities Scales of Personality (SSP) questionnaire were collected from 172 psychiatric outpatients and 46 healthy controls. The blood samples were analysed for plasma leptin, adiponectin, CRP, IL-6 and TNF-α. Associations between neuroticism and biomarkers were assessed using Spearman’s correlation coefficients and generalized linear models adjusting for confounders. In the adjusted generalized linear models, neuroticism predicted the leptin/adiponectin ratio (p = 0.003), leptin (p = 0.004) and IL-6 (p = 0.001). These associations were not better explained by current major depressive disorder and/or anxiety disorder. Adiponectin, CRP and TNF-α were not associated with neuroticism. In conclusion, the findings suggest that high neuroticism is related to elevated levels of plasma leptin/adiponectin ratio, leptin and IL-6 in young adults. Young adults with high neuroticism may therefore benefit from preventive interventions to decrease the risk for future metabolic and cardiovascular morbidity, but more research is required to test this hypothesis.

## Introduction

Personality traits reflect an individual’s consistent patterns of thoughts, feelings and behaviour and are important for how an individual will respond to and interact with his or her environment^1^. Enduring personality traits, such as neuroticism, may confer long-term vulnerability for shifts in biological homeostasis. These shifts may then over time contribute to the development of disease. Neuroticism is a major personality trait defined by frequent and intense experiences of negative emotions (e.g. anxiety), sensitivity to stress and a predisposition to perceive situations as threatening^2,3^. High levels of neuroticism are associated with common psychopathology, like depression and anxiety disorders^2^, as well as physical health problems. High neuroticism has been suggested as a possible risk factor for cardiovascular disease (CVD)^4,5^. It may also have synergistic effects on the increased risk for CVD in individuals with major depressive disorder (MDD)^4^. A large prospective cohort study reports a 12% increased risk for CVD mortality for a one standard deviation increase in neuroticism score^5^. Another large prospective cohort study describes a 40% increased risk specifically for ischemic heart disease mortality, in the group with the highest degree of neuroticism^6^. Growing evidence, although inconclusive, also suggest that high neuroticism is associated with metabolic and immune dysregulation^7–9^.

The underlying mechanism explaining the association between neuroticism and CVD remains unknown. However, the literature suggests that socioeconomic status and life-style behaviours may contribute^10,11^. Similar to depression, high neuroticism is associated with an unhealthy lifestyle^2,3,12^. Neuroticism traits may increase the likelihood that a person experiences psychosocial stress or engages in unhealthy life-style behaviours (e.g. substance use, physical inactivity, poor diet or smoking)^2,3^. Harmful behaviours and stress may in turn contribute both directly and indirectly to cardiovascular, metabolic, endocrine or immune dysregulation related to risk for CVD pathogenesis^12^.

Several proteins involved in immune regulation and adipose tissue function are considered potential biomarkers of cardiovascular and metabolic disease risk^13^. Out of these, C-reactive protein (CRP), Interleukin (IL)-6, Tumour necrosis factor (TNF)-α, leptin and adiponectin may be of particular interest in the context of neuroticism, given their association with internalizing problems. Elevated levels of CRP, IL-6 and TNF-α have all been robustly associated with MDD^14,15^ and growing evidence indicates a similar link to anxiety disorders^16,17^. Previous studies also indicate an association between MDD and elevated levels of leptin and decreased levels of adiponectin, but the evidence is still inconsistent^18–20^.

Elevated levels of CRP, IL-6 and TNF-α indicate chronic low-grade inflammation, which has a key role in the development of atherosclerosis^21^. CRP, IL-6 and TNF-α levels also predict cardiovascular morbidity and mortality^22,23^. Leptin and adiponectin are adipocyte-derived hormones linked to the metabolic syndrome^13^. Leptin is involved in the long-term regulation of energy balance, the hypothalamic–pituitary–adrenal (HPA)-axis and the immune system^24^. High leptin levels can unfavourably affect the vascular structure by promoting hypertension, angiogenesis and atherosclerosis and are a risk factor for myocardial infarction^13^. In contrast to leptin, adiponectin has protective anti-inflammatory, anti-atherogenic and insulin-sensitizing effects^13^. Low levels of adiponectin are a risk factor for diabetes type 2 and CVD^13,25^. The leptin-adiponectin (L/A) ratio is suggested as a sensitive biomarker for early metabolic dysregulation (e.g. changes in insulin sensitivity and triglyceride clearance), and may be more useful than each alone^26^.

Some studies have investigated if neuroticism is associated with inflammatory serum markers like CRP or cytokines, predominantly IL-6, with mixed results^8,10,27–36^. In a recent meta-analysis, no associations were found between neuroticism and CRP or IL-6^8^. This could partly be explained by the heterogeneity between previous studies, with differences in sample size and quality and content of the personality questionnaires^8^.

Far less work has addressed a potential association between neuroticism and leptin, adiponectin, or the L/A ratio. While Narita and colleagues observe a positive correlation between trait anxiety and the L/A ratio in healthy elders^37^, others have not found any association between neuroticism and leptin or adiponectin in community-dwelling adults or women with severe, morbid obesity^27,38^. None of these studies have looked into individuals with psychiatric disorders, who may be a risk population for maladaptive neuroticism.

To summarize, there is a paucity of studies examining the link between neuroticism and early biomarkers of metabolic and immune dysregulation, especially in young adults and risk populations for maladaptive neuroticism. Previous studies have also showed mixed results. Given the negative consequences of metabolic and immune dysregulation on the risk for CVD, early detection is important in risk populations such as patients with psychiatric disorders.

Using a cohort of young adults seeking psychiatric care and healthy age-matched controls, we saw an opportunity to approach this research question. Young adults seeking psychiatric care were considered an appropriate study population since common psychiatric disorders are linked to higher neuroticism^2^, psychiatric patients are a risk population for cardiovascular and metabolic disease^15,39^ and metabolic dysregulation in young adulthood may have far-reaching effects on long-term health.

The majority of the young adults seeking psychiatric outpatient care in Uppsala are women with affective or anxiety disorders^40^. Women may be of particular interest in the context of metabolic/immunological dysregulation and neuroticism, since they generally report a higher degree of neuroticism^2^ and have distinctively higher circulating levels of leptin^41^ compared with men.

An unhealthy life-style (e.g. smoking and substance addiction), affective disorders and use of antidepressants may be confounding or mediating factors for the relationship between early biomarkers of metabolic and cardiovascular risk and neuroticism, since they are linked to both^2,18,42,43^. Some established risk factors for metabolic and cardiovascular disease (e.g. elevated blood pressure) are associated with neuroticism^9,44^, but could be considered manifestations of the same disease process, rather than confounders.

We hypothesized that neuroticism is (i) positively associated with the L/A ratio, leptin, CRP, IL-6, TNF-α and (ii) negatively associated with adiponectin. Because personality traits may have more long-term effects on somatic health than state conditions, due to their persistence over time, we also hypothesized that (iii) neuroticism is a stronger predictor of these markers than a state diagnosis of MDD and/or anxiety disorder. Because both leptin and adiponectin levels are higher in women and women are overrepresented in psychiatric disease, we further hypothesized that (iv) these associations are stronger in women than in men.

## Methods

### Study design and participants

The material and data used in this cross-sectional study were obtained from the Uppsala Psychiatric Patient Samples (UPP), which has previously been described in more detail^45,46^. The UPP cohort was initiated in October 2012 and all consecutive new patients aged 18 to 25 years at the psychiatric outpatient clinic for young adults at the Department of Psychiatry in Uppsala, Sweden, are asked to participate. The patients primarily suffer from affective disorders, with varying stage and degree of illness. The patient data in this study were gathered between 2012 and 2014. Since 2013, individuals without previous or current contact with psychiatry units were recruited to the UPP from a pool of university students and personnel and included as a control cohort. The control data and samples comprising this study were collected between 2013 and 2015.

Patients were included in the study if they were between 18 and 25-years-old and seeking psychiatric outpatient-care. Controls were included if they were aged 18 to 30. Controls who screened positive for a current psychiatric disorder (n = 8) in the diagnostic interviews (see more details below) or had current psychotropic medication (n = 3) were excluded from the study.

Exclusion criteria for the total study population were systemic inflammatory disease (n = 9), diabetes mellitus (n = 3), cancer (n = 0), coeliac disorder (n = 7), pregnancy (n = 1), treatment with testosterone (n = 1), lack of blood sample or insufficient diagnostic assessments (n = 15), incomplete neuroticism score (n = 6), current diagnosis of bulimia nervosa (n = 16) or anorexia nervosa (n = 3) or if more than 4 months had passed between the initial health examination and blood sample collection (n = 1). The median time between health examination and blood sample collection was 11 days (IQR = 19 days). In total, 228 patients (179 women and 49 men) and 60 healthy controls (45 women and 15 men) had data available from the UPP during the study period. After exclusion criteria were applied, 173 patients (133 women and 40 men) and 46 controls (36 women and 10 men) remained and were included in the study (see Supplementary Fig. S1 for a flow-chart of the selection process). One patient without a previous history of diabetes was later excluded when it was discovered that she had a HbA1c value of 67, thereby fulfilling the diagnostic criteria for diabetes. Out of the remaining 218 participants, 46 were healthy controls and 172 were psychiatric patients, Table 1.

**Table 1:** Descriptive table of the study participants and comparisons between psychiatric patients and healthy controls.

	Total study population (N = 218)	Healthy controls (n = 46)	Psychiatric patients (n = 172)
**General characteristics**			
Sex: Female, n (%)	168 (77)	36 (78)	132 (77)
Age, years, median (IQR)	21.00 (3.00)	22.00 (3.00)	21.00 (4.00)***
Smoking, n (%)^b^	52 (24)	1 (2)	51 (30)***
Educational level: University, n (%)	127 (58)	45 (98)	82 (48)***
**Psychiatric characteristics**			
Neuroticism score, mean (SD)	59.10 (8.75)	50.39 (5.92)	61.43 (7.88)***
Antidepressants, n (%)^a^	82 (38)	0 (0)	82 (48)***
Any anxiety disorder, n (%)	108 (50)	0 (0)	108 (63)***
Current MDD, n (%)	74 (34)	0 (0)	74 (43)***
Life-time unipolar depressive episode, n (%)	124 (57)	3 (7)	121 (70)***
Bipolar disorder, n (%)	27 (12)	0 (0)	27 (16)**
Substance addiction, n (%)	16 (7)	0 (0)	16 (9)*
**Somatic status**			
BMI, kg/m^2^, median (IQR)^c^	22.41 (4.80)	22.38 (2.67)	22.49 (5.63)
WHR, median (IQR)^d^	0.76 (0.08)	0.74 (0.07)	0.76 (0.08)**
SBP, mmHg, median (IQR)^e^	119.00 (10.00)	110.00 (15.00)	120.00 (10.00)*
DBP, mmHg, median (IQR)^e^	70.00 (5.00)	70.00 (10.00)	65.50 (5.00)*
**Biomarkers of interest**			
L/A ratio, median (IQR)	1.35 (2.09)	1.17 (1.19)	1.47 (2.37)
Leptin, ng/mL, median (IQR)	14.00 (17.46)	11.48 (12.13)	16.31 (20.85)*
Adiponectin, ug/mL, median (IQR)	9.45 (4.49)	9.36 (4.09)	9.45 (4.69)
CRP, mg/L, median (IQR)^f^	0.79 (2.10)	0.46 (0.64)	1.00 (2.51)*
IL-6, pg/mL, median (IQR)^f^	0.29 (0.34)	0.23 (0.22)	0.33 (0.37)**
TNF-α, pg/mL, median (IQR)^f^	1.86 (1.02)	1.91 (0.97)	1.85 (1.18)

To test if there was a significant difference between patients and controls, the Chi-square test (or Fisher’s test when applicable) was used for the categorical variables and the Mann–Whitney test for the continuous variables.

*IQR* interquartile range, *BMI* body mass index, *WH*R Waist-hip ratio, *SBP* systolic blood pressure, *DBP* diastolic blood pressure, *L/A* leptin/adiponectin.

*p < 0.05, **p < 0.01, ***p < 0.001.

^a^SSRI, SNRI, tricylic antidepressants, Mirtazapine, Mianserin or Buproprion.

Missing data for: ^b^Fourteen controls and forty-nine patients; ^c^Four patients; ^d^Thirteen patients and two controls; ^e^Two patients; ^f^Sixteen patients.

Participants first underwent an initial clinical health examination at the time of inclusion. Blood pressure was measured while seated. Weight was measured with a digital scale in kg and body-mass index (BMI) was calculated as weight in kg divided by height in squared meters. A waist-hip ratio was calculated from waist and hip circumference measurements. The participants returned for a second visit in which questionnaires on socio-demographics, medical history, current medication (including use of antidepressants) and smoking status (yes/no) were answered in conjunction with blood sample collection.

### Independent variables

Psychiatric diagnoses were assessed using the Diagnostic and Statistical Manual of Mental Disorders (DSM)-IV criteria^47^. The assessment was based on a clinical interview and a diagnostic interview with the M.I.N.I.-International Neuropsychiatric Interview (M.I.N.I. 6.0)^48^ or the Swedish version of the Structured Clinical Interview for DSM IV axis I disorders (SCID-I)^49^. The controls were interviewed with the M.I.N.I. 6.0.

Personality traits were assessed with the Swedish universities Scales of Personality (SSP) self-rating questionnaire. It has demonstrated good psychometric properties in a Swedish normative sample^50^. The SSP is rated on a four-point Likert scale (1 “does not apply at all” to 4 “applies completely”) with 91 items grouped into 13 different scales where each scale is composed of seven items^50,51^. The SSP is designed to assess personality traits linked to biological correlates and vulnerability to psychopathology, making it a relevant questionnaire in psychobiological research^50,51^.

SSP scale scores were transformed to standardized T-scores, with a computerized script in accordance with instructions in the Swedish SSP manual (version 2.1). The T-scores are adjusted for age and sex, and standardized to have a mean of 50 and a standard deviation (SD) of 10, based on a representative Swedish sample^50^. Factor analyses in previous studies suggest that the SSP scales measure three broader dimensions of personality: neuroticism, extraversion and aggression^50,51^. These factors correlate with the basic personality dimensions of the Revised NEO Personality Inventory scales, according to an Estonian study^51^. A neuroticism score was constructed from the overall mean of the T-scores of the SSP scales constituting the neuroticism factor in previous studies, i.e. Somatic trait anxiety, Psychic trait anxiety, Stress susceptibility, Lack of assertiveness, Embitterment and Mistrust^50,51^. Gustavsson and colleagues reported a satisfactory internal consistency for these SSP subscales, with Cronbach’s alpha coefficients ranging from 0.74 to 0.82^50^. A higher neuroticism score indicates a higher degree of neuroticism.

### Dependent variables

#### Blood sample collection and assessment of biomarkers

Blood samples were obtained non-fasting. Samples were collected during office hours (mean = 12:00 AM, SD = 2 h) and kept in − 80 °C at Uppsala Biobank. Total plasma adiponectin and leptin were analysed with a solid phase sandwich enzyme-linked immunosorbent assay (ELISA) (Mercodia AB, Uppsala, Sweden). This procedure is previously described in more detail^45^. Assay sensitivity was 0.05 ng/mL for leptin and 1.25 ng/mL for adiponectin. Three controls and two patients had undetectable measures for leptin, which was replaced by the lowest limit of detection. The total assay mean coefficient of variation was 5.6% for adiponectin and 5.0% for leptin.

Plasma protein levels of CRP, IL-6 and TNF-α were analysed with an electrochemiluminescence sandwich immunoassay using Meso Scale Discovery (K15049D and K15198D, Rockville, MD, USA) multiplex platform. According to the manufacturer, the inter-assay variation for CRP, IL-6 and TNF-α was less than 10%.

The plasma leptin concentration (ng/mL) was divided by the plasma adiponectin concentration (ug/mL) to create the L/A ratio.

### Confounders

Sex, use of antidepressants, current MDD, substance addiction and anxiety disorders were considered possible confounders. The MDD, substance addiction and anxiety disorder diagnoses were based on the results of the clinical and diagnostic interviews. The anxiety disorder diagnoses included generalized anxiety disorder, social anxiety disorder, panic disorder, agoraphobia, post-traumatic stress disorder or obsessive–compulsive disorder. Smoking was considered a possible confounder but not included in the statistical analysis due to lack of data in the male study population.

### Statistics

Data were analysed using the Statistical Package for the Social Sciences (SPSS v25, IBM). Statistical significance was defined as p < 0.05. Data were regarded as normally distributed if the following conditions were met: skewness divided by the standard error of skewness between ± 2 and visually estimated normal distribution (histogram, box-plot). None of the dependent variables (L/A ratio, leptin, adiponectin, CRP, IL-6 and TNF-α) were normally distributed.

To test if there was a significant difference in psychiatric characteristics, life-style factors, somatic status or biomarkers of interest between patients and controls, the Chi-square test (or Fisher’s test when appropriate) was used for the categorical variables and the Mann–Whitney test for the continuous variables.

Associations between the neuroticism score and the dependent variables (L/A ratio, leptin, adiponectin, CRP, IL-6 and TNF-α), BMI, waist-hip ratio and systolic- and diastolic blood pressure were assessed with Spearman’s correlation coefficients. For the dependent variables associated with neuroticism, circulating levels were compared between participants with a high neuroticism score (1 SD or more above the norm) and a low-normative neuroticism score (below the norm) with the Mann–Whitney test. The effect size for the group comparisons was estimated with Rosenthal’s effect size ((r = z/(SQRT(n))))^52^. Because of known large differences in plasma leptin levels between men and women^45^, we repeated the Spearman’s correlation tests and the group comparisons in men and women separately.

Generalized linear models (GLzM) with gamma log link distribution were constructed for each dependent variable that was significantly associated with the neuroticism score in the Spearman’s test. The neuroticism score was included in the GLzMs as an independent variable along with possible confounders. The generalized linear models were not adjusted for biological variables (e.g. BMI, waist-hip ratio and blood pressure) known to impact the dependent variables, if they could also be considered part of the same metabolic/cardiovascular dysregulation process as the dependent variables. Instead, these biological variables were included in a generalized linear model as the dependent variable, if they were significantly associated with the neuroticism score in the Spearman’s test.

As sensitivity analyses, the GLzMs were repeated in the patient population (without controls) and in the female study population. The male study population was too small to perform GLzMs.

In an exploratory post-hoc analysis, Spearman’s bivariate correlation coefficients were also used to explore the associations between the T-scores of the SSP subscales constituting the neuroticism score and the L/A ratio, leptin, adiponectin, CRP, IL-6 and TNF-α.

### Ethics

The study was carried out in accordance with the latest version of the Declaration of Helsinki and was approved by the Regional Ethical Review Board in Uppsala, Sweden. Fully informed and written consent was obtained from each participant. Overall, patients participating in the UPP report a positive response to participation in research, with high voluntariness and low levels of regret^46^.

## Results

### Participant characteristics

Characteristics of the study sample and comparisons between psychiatric patients and healthy controls are presented in Table 1. A majority were women. Fifty-eight percent reported university studies as their highest level of education. Eighteen percent were obese.

The psychiatric patients reported higher neuroticism scores than the controls (p < 0.001). They were also younger and less likely to have attended university. The waist-hip ratio was higher in the group with psychiatric patients than in the group with healthy controls. However, there was no difference in BMI between the groups. Furthermore, the psychiatric patients had higher systolic blood pressure, but lower diastolic blood pressure than the controls. In addition, plasma leptin, CRP and IL-6 levels were higher in the psychiatric patients than in the controls. The L/A ratio and adiponectin and TNF-α levels did not differ between the groups.

### Associations between the study variables

The results of the Spearman’s correlation tests are presented in Table 2. The neuroticism score was positively correlated with the L/A ratio, leptin and IL-6. Adiponectin, CRP and TNF-α were not correlated with the neuroticism score. The neuroticism score was also positively correlated with the waist-hip ratio, but not with BMI, systolic blood pressure or diastolic blood pressure. In the female study population, the neuroticism score remained correlated with the L/A ratio (r = 0.26, p = 0.001), leptin (r = 0.27, p = 0.0004) and IL-6 (r = 0.20, p = 0.011). No correlation was found between the neuroticism score and any of the dependent variables in the male study population, Supplementary Table S1.

**Table 2:** Spearman’s correlation coefficients (ρ) in the total study population (N = 218).

	Neuroticism score	L/A ratio
L/A ratio	0.19**	–
Leptin	0.19**	0.94***
Adiponectin	− 0.05	− 0.30***
CRP^a^	0.11	0.40***
IL-6^a^	0.18*	0.31***
TNF-α^a^	− 0.05	− 0.04
BMI^b^	0.12	0.55***
WHR^c^	0.15*	− 0.03
SBP^d^	0.06	0.27***
DBP^d^	− 0.04	0.31***

*BMI* body mass index, *WHR* waist-hip ratio, *SBP* systolic blood pressure, *DBP* diastolic blood pressure, *L/A* leptin/adiponectin.

*p < 0.05; **p < 0.01; ***p < 0.001.

Missing data for: ^a^Sixteen patients; ^b^Four patients; ^c^Thirteen patients and two controls; ^d^Two patients.

**Figure 1:**
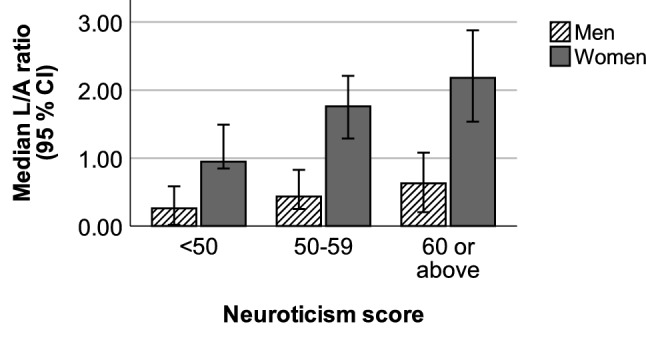
Leptin/adiponectin (L/A) ratio and neuroticism score in the female and male study populations.

In the total study population, the group with a high (≥ 60) neuroticism score had higher L/A ratio (n_1_ = 109, median = 1.59) than the group with a low-normative (< 50) neuroticism score (n_2_ = 34, median = 0.89; p = 0.002). Leptin levels were also higher in the high neuroticism group (n_1_ = 109, median = 17.01 ng/mL) than in the low-normative neuroticism score group (n_2_ = 34, median = 11.03 ng/mL, p = 0.002). IL-6 levels did not differ between the two groups (p = 0.07). Similarly, in the female study population, the group with a high neuroticism score had higher L/A ratio (n_1_ = 88, median = 2.18) than the low-normative neuroticism score group (n_2_ = 28, median = 0.95; p < 0.001), Fig. 1. The women with a high neuroticism score also had higher leptin levels (n_1_ = 88, median = 21.78 ng/mL) than the women in the low-normative neuroticism score group (n_2_ = 28, median = 11.92 ng/mL p < 0.001). In the male study population, there was a similar trend, but it was not significant, Fig. 1. In the total study population, the effect size was small for both the L/A ratio (r = 0.26) and the leptin levels (r = 0.25). In the female study population, the effect size was moderate for both the L/A ratio (r = 0.31) and the leptin levels (r = 0.33).

### Generalized linear models

In the generalized linear models (GLzMs) adjusting for possible confounders, the neuroticism score remained positively associated with the L/A ratio, leptin and IL-6, Table 3. Furthermore, the use of antidepressants was independently positively associated with the L/A ratio. Substance addiction was independently negatively associated with IL-6. MDD or any anxiety disorder were not associated with the L/A ratio, leptin or IL-6 in the GLzMs in the total study population, Table 3. The associations between the neuroticism score and the L/A ratio, leptin and IL-6 remained significant in the patient population (w/o controls) and in the female study population, Supplementary Tables S2 and S3. In the patient population, current MDD was negatively associated with the L/A ratio.

**Table 3:** Generalized linear models for the L/A ratio, leptin and IL-6 in the total study population (N = 218).

Factors	L/A ratio^a^	Leptin^a^	IL-6^b^
	B (SE)	B (SE)	B (SE)
Neuroticism score	0.023 (0.008)**	0.019 (0.007)**	0.023 (0.007)**
MDD	− 0.272 (0.140)	− 0.130 (0.122)	− 0.057 (0.128)
Any anxiety disorder	0.044 (0.147)	0.012 (0.126)	− 0.163 (0.132)
Antidepressants	0.435 (0.132)*	0.356 (0.115)**	0.100 (0.119)
Substance addiction	− 0.385 (0.237)	− 0.188 (0.207)	− 0.493 (0.222)*
Sex^c^	1.346 (0.146)***	1.538 (0.128)***	0.147 (0.137)

*L/A* leptin/adiponectin, *MDD* major depressive disorder, *SE* standard error.

*p < 0.05; **p < 0.01; ***p < 0.001.

Included: ^a^n = 218; ^b^n = 202; ^c^Reference = Male.

### Exploratory post-hoc analyses of the SSP subscales

In the total study population, the L/A ratio and leptin levels were both positively correlated with the SSP subscales Stress susceptibility, Mistrust and Embitterment. Adiponectin was also negatively correlated with Stress susceptibility, CRP was positively correlated with Embitterment and IL-6 was positively correlated with Stress susceptibility and Embitterment. Additional data are given in Supplementary Table S4.

In the female study population, the L/A ratio and leptin levels were positively correlated with Psychic trait anxiety, Somatic trait anxiety, Stress susceptibility, Mistrust and Embitterment. Adiponectin was negatively correlated with Stress susceptibility. CRP was positively correlated with Somatic trait anxiety. IL-6 was positively correlated with Somatic trait anxiety and Stress susceptibility. None of the SSP subscales included in the neuroticism score were correlated with the dependent variables in the male study population. Additional data are given in Supplementary Table S5.

## Discussion

**Figure 2:**
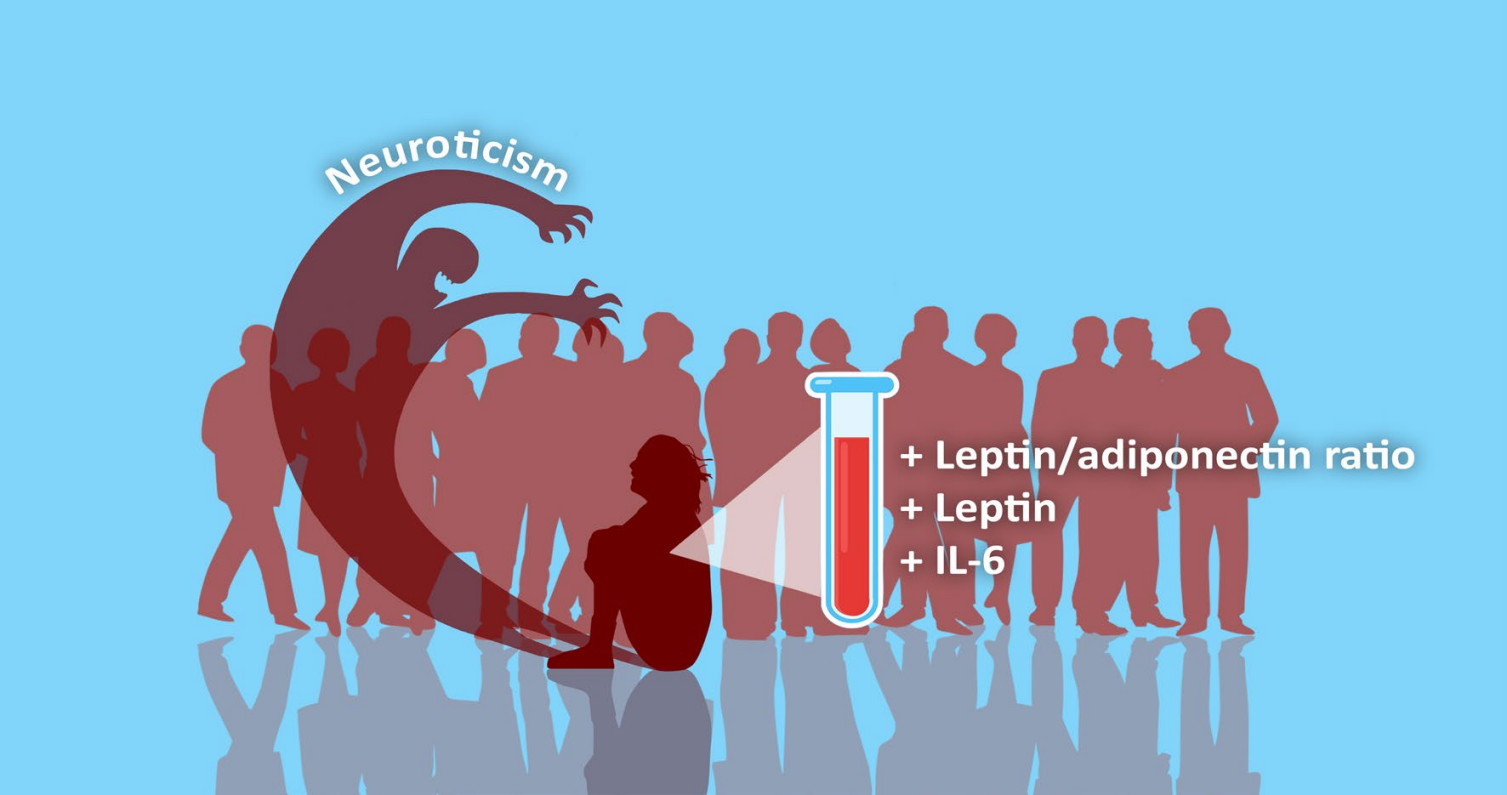
Graphical abstract.

This study aimed to investigate the relationship between neuroticism and biomarkers involved in immune system/adipose tissue crosstalk, with relevance for metabolic and cardiovascular disease risk, in young adults with and without psychiatric co-morbidity. Out of these biomarkers, neuroticism was positively associated with the L/A ratio, leptin and IL-6, independent of current diagnosis of MDD or anxiety disorder. The degree of neuroticism was not related to circulating levels of adiponectin, CRP and TNF-α. See Fig. 2 for a graphical abstract of the study.

To the best of our knowledge, this is the first study to report an association between neuroticism and leptin. This study also replicates and extends the literature on the relationship between neuroticism, the L/A ratio, adiponectin, CRP, IL-6 and TNF-α by focusing on previously unaddressed populations: young adults with and without psychiatric disorders. Elevated L/A ratio, elevated leptin and decreased adiponectin are suggested early biomarkers of the metabolic syndrome^13^. The metabolic syndrome may in turn contribute to the elevated risk for cardiovascular morbidity and early death observed in psychiatric patients^15,39^. In this context, the focus on young psychiatric patients is of extra importance because it is a patient population for whom early preventive measures and treatment may have a large impact on their future health.

The positive association between neuroticism and L/A ratio is in line with the results of Narita et al. observing a positive association between trait anxiety and L/A ratio. In contrast to the findings of our study, three previous studies observe no significant associations between leptin and neuroticism or trait anxiety in non-psychiatric populations^27,37,38^. Potential explanations include between-studies differences in age, choice of personality questionnaire, neuroticism range, socioeconomic status, somatic and mental health state and methods for blood sample collection/biomarker analysis. For example, non-fasting blood samples were used in the present study, whereas previous studies of personality and leptin used fasting blood samples. The study population of our study also reported relatively high levels of neuroticism, which are likely to be associated with a higher degree of maladaptive biological functioning and behaviour. Considering this, it can be speculated that one possible reason some previous studies did not observe an association between leptin and neuroticism, is that their non-psychiatric study populations had neuroticism levels closer to the norm, which may not be as maladaptive. Neuroticism may in some individuals even have beneficial health effects^3^, which could contribute to heterogeneity between studies.

Blunted cortisol and cardiac stress reactivity may be related to high neuroticism^53^. This could possibly explain our observation of a positive association between neuroticism and plasma leptin levels, because decreased sympathetic activity increases leptin synthesis and secretion from adipose tissue in animal models and cortisol levels are negatively associated with leptin levels^42,54^. Life-style factors may also contribute to the positive association between neuroticism and leptin levels, but this was not supported by the available data in our study.

Leptin administration in animals have shown some promise for anxiety reduction^55–63^, which would seem to be in contrast with our finding of a positive association with neuroticism. One possible explanation for this discrepancy is that higher plasma leptin might not reflect increased leptin signalling in the central nervous system, because of central leptin resistance^64^. It is also still unclear how results in animals are attributable to the administrated dose of leptin in relation to physiological levels or to alterations in downstream regulation of leptin signalling.

Neuroticism was positively associated with IL-6, but not with CRP or TNF-α. Some previous studies also observe a positive association between IL-6 and neuroticism^29,34,35^, whereas others observe a negative association^30,65^ or no association^8,66,67^. Elliot et al. (2017) observe a positive association with IL-6 in a subgroup with low socioeconomic status, but a negative association in a subgroup with high socioeconomic status. Differences in socioeconomic status are therefore one possible explanation for the mixed results between studies. Most previous work support our result that neuroticism was not associated with CRP^8,28,31–33,68^ or TNF-α^28,32^. Studies observing a positive association between CRP and neuroticism^35,69^ have a higher mean age than our study population, which could possibly explain the difference in results. Because IL-6 upregulation is not specific for an inflammatory state^70^, the lack of association with TNF-α suggests that the association between IL-6 and neuroticism may not be explained by low-grade inflammation, but by other mechanisms involving IL-6, e.g. energy homeostasis or tissue repair.

There were no significant associations between any of the dependent variables and the neuroticism score in the men. This could suggest that the associations are specific for women, but the results should be interpreted with much caution since the male study population was underpowered. Before any conclusions can be drawn regarding a possibly sex-specific association, the results need to be repeated and validated in a larger male study population.

An exploratory analysis with all the subscales of the neuroticism dimension in a post-hoc exploratory analysis showed that higher leptin and L/A ratio were linked to several of the constituent subscales. Similar to earlier research, there was no association between adiponectin and neuroticism in this study. However, based on the results of the exploratory analysis, it can be hypothesized that adiponectin is negatively associated with stress susceptibility specifically, but not with overall neuroticism. In support of this hypothesis, there are earlier observations of decreased adiponectin in stress-susceptible mice in response to chronic stress; mediated by a decrease in adipose peroxisome proliferator-activator receptor (PPAR)-y, a key regulator of adiponectin production^71^. Adiponectin is also suggested to have a protective effect against stress-induced negative effects on emotional homeostasis^71,72^, wherefore decreased adiponectin may contribute to increased stress-susceptibility.

Systolic or diastolic blood pressure were not related to neuroticism in our study population, which is in contrast with the results of previous studies^9,44^.

The findings of this study need to be considered in light of several limitations. Because of the cross-sectional design the study cannot be used to infer conclusions on causality. Prospective studies should therefore be considered as a next step in future research. Another major limitation is the use of non-fasting blood samples. However, there is a high correlation (sex-adjusted partial correlation coefficients ≥ 0.95) between fasting and non-fasting blood-samples for leptin, adiponectin and the L/A ratio^73^. The choice of study population limits the generalizability of the results (e.g. to a different age distribution, or non-psychiatric populations), but reduces the risk that the observed effects are influenced by age. Furthermore, it cannot be excluded that the psychiatric state of the participants (e.g. co-morbid depressive symptoms) may have influenced their response to the SSP questionnaire^2^. In future studies, this potential bias could possibly be addressed by including repeated measurements, multiple informants or clinical evaluations. Although we controlled the results for some potential confounders, data were not available on other important possible confounders such as physical activity or diet^3,30,42,74^. The small sample size may have limited the power to detect significant effects, especially in the male study population. The null associations in the men should therefore be interpreted with caution. Finally, it is a limitation that other personality traits relevant to both psychopathology and cardio-metabolic health were not measured in this study.

Strengths of the present study include a study population with a wide range of neuroticism, a panel of multiple biomarkers of inflammation and adipose tissue dysfunction, systematic and standardized diagnostic assessments for all participants and the use of a questionnaire for assessment of neuroticism that has been designed to assess traits related to psychopathology and validated for a Swedish population.

In conclusion, the findings suggest that higher neuroticism, independent of diagnosis of current MDD or anxiety disorder, is associated with elevated levels of L/A ratio, leptin and IL-6 in young adults with and without psychiatric co-morbidity. We demonstrate that these associations are prevalent already in early adulthood and this is in line with the hypothesis that high neuroticism may be a vulnerability factor for future metabolic and cardiovascular disease risk. Young adults with psychiatric disorders and high neuroticism may therefore benefit from interventions to prevent future metabolic and cardiovascular morbidity, but more research is required to test this hypothesis. These findings merit further study in other populations, preferably with a prospective study design to provide more information on causality.

## Supplementary Information

Supplementary Information.

## Data Availability

The data that support the findings of this study are available from the corresponding author upon request in accordance with the General Data Protection Regulation.

## References

[1] Diener E, Lucas RE (2020). Personality Traits.

[2] Lahey BB (2009). Public health significance of neuroticism. Am. Psychol..

[3] Mutambudzi M, Flowers P, Demou E (2019). Emergency personnel neuroticism, health and lifestyle: A UK Biobank study. Occup. Med. (Lond.).

[4] Almas A, Moller J, Iqbal R, Forsell Y (2017). Effect of neuroticism on risk of cardiovascular disease in depressed persons—A Swedish population-based cohort study. BMC Cardiovasc. Disord..

[5] Shipley BA, Weiss A, Der G, Taylor MD, Deary IJ (2007). Neuroticism, extraversion, and mortality in the UK Health and Lifestyle Survey: A 21-year prospective cohort study. Psychosom. Med..

[6] Narita M, Tanji F, Tomata Y, Mori K, Tsuji I (2020). The mediating effect of life-style behaviors on the association between personality traits and cardiovascular disease mortality among 29,766 community-dwelling Japanese. Psychosom. Med..

[7] Mommersteeg PM, Pouwer F (2012). Personality as a risk factor for the metabolic syndrome: A systematic review. J. Psychosom. Res..

[8] Luchetti M, Barkley JM, Stephan Y, Terracciano A, Sutin AR (2014). Five-factor model personality traits and inflammatory markers: New data and a meta-analysis. Psychoneuroendocrinology.

[9] Sutin AR, Stephan Y, Terracciano A (2019). Personality and metabolic dysfunction in young adulthood: A cross-sectional study. J. Health Psychol..

[10] Elliot AJ, Turiano NA, Chapman BP (2017). Socioeconomic status interacts with conscientiousness and neuroticism to predict circulating concentrations of inflammatory markers. Ann. Behav. Med..

[11] Hagger-Johnson G (2012). Neuroticism and cardiovascular disease mortality: Socioeconomic status modifies the risk in women (UK Health and Lifestyle Survey). Psychosom. Med..

[12] Penninx BW (2017). Depression and cardiovascular disease: Epidemiological evidence on their linking mechanisms. Neurosci. Biobehav. Rev..

[13] Srikanthan K, Feyh A, Visweshwar H, Shapiro JI, Sodhi K (2016). Systematic review of metabolic syndrome biomarkers: A panel for early detection, management, and risk stratification in the west Virginian population. Int. J. Med. Sci..

[14] Dowlati Y (2010). A meta-analysis of cytokines in major depression. Biol. Psychiatry.

[15] Penninx B, Lange SMM (2018). Metabolic syndrome in psychiatric patients: Overview, mechanisms, and implications. Dialog. Clin. Neurosci..

[16] Michopoulos V, Powers A, Gillespie CF, Ressler KJ, Jovanovic T (2017). Inflammation in fear- and anxiety-based disorders: PTSD, GAD, and beyond. Neuropsychopharmacology.

[17] Naude PJW, Roest AM, Stein DJ, de Jonge P, Doornbos B (2018). Anxiety disorders and CRP in a population cohort study with 54,326 participants: The LifeLines study. World J. Biol. Psychiatry.

[18] Carvalho AF (2014). Adipokines as emerging depression biomarkers: A systematic review and meta-analysis. J. Psychiatr. Res..

[19] Milaneschi Y, Lamers F, Bot M, Drent ML, Penninx BW (2017). Leptin dysregulation is specifically associated with major depression with atypical features: Evidence for a mechanism connecting obesity and depression. Biol. Psychiatry.

[20] Cao B (2018). Leptin and adiponectin levels in major depressive disorder: A systematic review and meta-analysis. J. Affect. Disord..

[21] Libby P, Ridker PM, Maseri A (2002). Inflammation and atherosclerosis. Circulation.

[22] Singh-Manoux A (2017). Association between inflammatory biomarkers and all-cause, cardiovascular and cancer-related mortality. CMAJ.

[23] Kaptoge S (2014). Inflammatory cytokines and risk of coronary heart disease: New prospective study and updated meta-analysis. Eur. Heart J..

[24] Friedman JM (2019). Leptin and the endocrine control of energy balance. Nat. Metab..

[25] Kishida K, Funahashi T, Shimomura I (2014). Adiponectin as a routine clinical biomarker. Best Pract. Res. Clin. Endocrinol. Metab..

[26] Larsen MA (2018). Leptin to adiponectin ratio—A surrogate biomarker for early detection of metabolic disturbances in obesity. Nutr. Metab. Cardiovasc. Dis..

[27] Capuron L (2011). Relationship between adiposity, emotional status and eating behaviour in obese women: Role of inflammation. Psychol. Med..

[28] Wagner EN (2019). Associations of personality traits with chronic low-grade inflammation in a Swiss community sample. Front. Psychiatry.

[29] Sutin AR (2010). High neuroticism and low conscientiousness are associated with interleukin-6. Psychol. Med..

[30] Graham EK (2018). Physical activity mediates the association between personality and biomarkers of inflammation. SAGE Open Med..

[31] Sararoudi RB (2014). Is there any association of personality traits with vascular endothelial function or systemic inflammation?. Adv. Biomed. Res..

[32] Schmidt FM (2018). Serum markers of inflammation mediate the positive association between neuroticism and depression. Front. Psychiatry.

[33] Allen MS, Laborde S (2017). Five factor personality traits and inflammatory biomarkers in the English longitudinal study of aging. Person. Individ. Differ..

[34] FitzGerald L, Macey PM, Brecht M-L (2014). Personality, sex and systemic inflammation. Psychology.

[35] Millar K (2013). Personality, socio-economic status and inflammation: Cross-sectional, population-based study. PLoS ONE.

[36] Mwendwa DT (2013). Dispositional depression and hostility are associated with inflammatory markers of cardiovascular disease in African Americans. Brain Behav. Immun..

[37] Narita K (2008). Associations between trait anxiety, insulin resistance, and atherosclerosis in the elderly: A pilot cross-sectional study. Psychoneuroendocrinology.

[38] Sutin AR (2013). Personality traits and leptin. Psychosom. Med..

[39] Correll CU (2017). Prevalence, incidence and mortality from cardiovascular disease in patients with pooled and specific severe mental illness: A large-scale meta-analysis of 3,211,768 patients and 113,383,368 controls. World Psychiatry.

[40] Ramirez A, Ekselius L, Ramklint M (2009). Mental disorders among young adults self-referred and referred by professionals to specialty mental health care. Psychiatr. Serv..

[41] Ahima RS, Osei SY (2004). Leptin signaling. Physiol. Behav..

[42] Margetic S, Gazzola C, Pegg GG, Hill RA (2002). Leptin: A review of its peripheral actions and interactions. Int. J. Obes Relat. Metab. Disord..

[43] Archer M (2018). The effects of adiposity and alcohol use disorder on adipokines and biomarkers of inflammation in depressed patients. Psychiatry Res..

[44] van Reedt DAK, Giltay EJ, van Veen T, Zitman FG, Penninx BW (2012). Personality traits and childhood trauma as correlates of metabolic risk factors: The Netherlands study of depression and anxiety (NESDA). Prog. Neuropsychopharmacol. Biol. Psychiatry.

[45] Syk M (2018). Plasma levels of leptin and adiponectin and depressive symptoms in young adults. Psychiatry Res..

[46] Cunningham JL, Zanzi M, Willebrand M, Ekselius L, Ramklint M (2017). No regrets: Young adult patients in psychiatry report positive reactions to biobank participation. BMC Psychiatry.

[47] American Psychological Association (2000). Diagnostic and Statistical Manual of Mental Disorders: DSM-IV-TR.

[48] Sheehan DV (1998). The mini-international neuropsychiatric interview (M.I.N.I.): The development and validation of a structured diagnostic psychiatric interview for DSM-IV and ICD-10. J. Clin. Psychiatry.

[49] First MSR, Gibbon M, Williams J (1996). Structured Clinical Interview for DSM-IV Axis I Disorders, Clinician Version (SCID-CV).

[50] Gustavsson JP (2000). Swedish universities scales of personality (SSP): Construction, internal consistency and normative data. Acta Psychiatr. Scand..

[51] Aluoja A (2009). Personality traits measured by the Swedish universities scales of personality: Factor structure and position within the five-factor model in an Estonian sample. Nord J. Psychiatry.

[52] Rosenthal R, Cooper H, Hedges L (1994). Parametric measures of effect size. Handb. Res. Synth..

[53] Bibbey A, Carroll D, Roseboom TJ, Phillips AC, de Rooij SR (2013). Personality and physiological reactions to acute psychological stress. Int. J. Psychophysiol..

[54] Caron A, Lee S, Elmquist JK, Gautron L (2018). Leptin and brain-adipose crosstalks. Nat. Rev. Neurosci..

[55] Alo R (2017). Role of leptin and orexin-A within the suprachiasmatic nucleus on anxiety-like behaviors in Hamsters. Mol. Neurobiol..

[56] Asakawa A (2003). Leptin treatment ameliorates anxiety in ob/ob obese mice. J. Diabetes Complic..

[57] Audira G (2018). Zebrafish mutants carrying leptin a (lepa) Gene deficiency display obesity, anxiety, less aggression and fear, and circadian rhythm and color preference dysregulation. Int. J. Mol. Sci..

[58] Finger BC, Dinan TG, Cryan JF (2010). Leptin-deficient mice retain normal appetitive spatial learning yet exhibit marked increases in anxiety-related behaviours. Psychopharmacology.

[59] Liu J (2010). Acute administration of leptin produces anxiolytic-like effects: A comparison with fluoxetine. Psychopharmacology.

[60] Liu J, Guo M, Lu XY (2015). Leptin/LepRb in the ventral tegmental area mediates anxiety-related behaviors. Int. J. Neuropsychopharmacol..

[61] Liu J, Perez SM, Zhang W, Lodge DJ, Lu XY (2011). Selective deletion of the leptin receptor in dopamine neurons produces anxiogenic-like behavior and increases dopaminergic activity in amygdala. Mol. Psychiatry.

[62] Wang W (2015). Leptin: A potential anxiolytic by facilitation of fear extinction. CNS Neurosci. Ther..

[63] Tyree SM, Munn RG, McNaughton N (2016). Anxiolytic-like effects of leptin on fixed interval responding. Pharmacol. Biochem. Behav..

[64] Martin SS, Qasim A, Reilly MP (2008). Leptin resistance: A possible interface of inflammation and metabolism in obesity-related cardiovascular disease. J. Am. Coll. Cardiol..

[65] Turiano NA, Mroczek DK, Moynihan J, Chapman BP (2013). Big 5 personality traits and interleukin-6: Evidence for "healthy Neuroticism" in a US population sample. Brain Behav. Immun..

[66] Chapman BP (2009). Gender, race/ethnicity, personality, and interleukin-6 in urban primary care patients. Brain Behav. Immun..

[67] Chapman BP (2011). Openness and conscientiousness predict 34-week patterns of Interleukin-6 in older persons. Brain Behav. Immun..

[68] Mottus R, Luciano M, Starr JM, Pollard MC, Deary IJ (2013). Personality traits and inflammation in men and women in their early 70s: The Lothian Birth Cohort 1936 study of healthy aging. Psychosom. Med..

[69] Armon G, Melamed S, Shirom A, Berliner S, Shapira I (2013). The associations of the Five Factor Model of personality with inflammatory biomarkers: A four-year prospective study. Person. Individ. Differ..

[70] Del Giudice M, Gangestad SW (2018). Rethinking IL-6 and CRP: Why they are more than inflammatory biomarkers, and why it matters. Brain Behav. Immun..

[71] Guo M (2017). Role of the adipose PPARgamma-adiponectin axis in susceptibility to stress and depression/anxiety-related behaviors. Mol. Psychiatry.

[72] Sun F (2019). Adiponectin modulates ventral tegmental area dopamine neuron activity and anxiety-related behavior through AdipoR1. Mol. Psychiatry.

[73] Hancox RJ, Landhuis CE (2011). Correlation between measures of insulin resistance in fasting and non-fasting blood. Diabetol. Metab. Syndr..

[74] Allen MS, Walter EE, McDermott MS (2017). Personality and sedentary behavior: A systematic review and meta-analysis. Health Psychol..

