# Supplementary material for: Neuroticism is positively associated with leptin/adiponectin ratio, leptin and IL-6 in young adults
Source: Sci Rep. 2021 May 7;11:9690. doi: 10.1038/s41598-021-89251-y (PMC8105321; doi:10.1038/s41598-021-89251-y)
Supplement: Supplementary file 1 — Supplementary Information. [file 41598_2021_89251_MOESM1_ESM.docx]

**Supplementary material**

**p 1 Table S1.** Spearman’s correlation coefficients (ρ) with separate analyses for women and men

**p 2 Table S2.** Generalized linear models for the L/A ratio, leptin and IL-6 in the patient population (N=172)

**p 3 Table S3.** Generalized linear models for the L/A ratio, leptin and IL-6 in the female study population (N=168)

**p 4 Table S4.** Spearman’s correlation coefficients for the neuroticism score and the neuroticism score subscales in the total study population (T) and the patient population (P)

**p 5 Table S5.** Spearman’s correlation coefficients for the neuroticism score and the neuroticism score subscales in the female study population (F) and the male study population (M)

**p 6 Figure S1.** Flow-chart of the selection process

**Table S1.** Spearman’s correlation coefficients (ρ) with separate analyses for women and men

|  | **Neuroticism score** | | **L/A ratio** | |
| --- | --- | --- | --- | --- |
|  | Women (n=168) | Men (n=50) | Women (n=168) | Men (n=50) |
| **L/A ratio** | 0.26 *** | 0.15 | - | - |
| **Leptin** | 0.27 *** | 0.14 | 0.93 *** | 0.93 *** |
| **Adiponectin** | -0.04 | <0.01 | -0.55 *** | -0.32 * |
| **CRP** ^a^ | 0.13 | 0.12 | 0.40 *** | 0.41 ** |
| **IL-6** ^a^ | 0.20 * | 0.12 | 0.36 *** | 0.28 |
| **TNF-α** ^a^ | -0.09 | 0.05 | 0.01 | <-0.01 |
| **BMI** ^b^ | 0.15 | <-0.01 | 0.67 *** | 0.50 *** |
| **WHR** ^c^ | 0.17 * | 0.19 | 0.36 *** | 0.26 |
| **SBP** ^d^ | 0.05 | 0.06 | 0.27 *** | -0.14 |
| **DBP** ^d^ | -0.02 | 0.10 | 0.31 *** | 0.14 |
| * p<0.05; ** p<0.01; *** p<0.001; BMI = Body mass index; WHR = Waist-hip ratio; SBP = Systolic blood pressure; DBP = Diastolic blood pressure; L/A = Leptin/adiponectin; Missing data for: ^a^ Nine female patients and 7 male controls; ^b^ Four female patients; ^c^ Nine female patients, two female controls and four male controls; ^d^ Two female patients. | | | | |

| **Table S2.** Generalized linear models for the L/A ratio, leptin and IL-6 in the patient population (N=172) | | | |
| --- | --- | --- | --- |
|  | **L/A ratio ^a^** | **Leptin ^a^** | **IL-6 ^b^** |
| Factors | B (SE) | B (SE) | B (SE) |
| **Neuroticism score** | **0.025 (0.009) **** | **0.018 (0.008) *** | **0.027 (0.008) **** |
| **MDD** | **-0.285 (0.138) *** | -0.156 (0.119) | -0.105 (0.128) |
| **Any anxiety disorder** | 0.003 (0.149) | -0.043 (0.127) | -0.271 (0.138) |
| **Antidepressants** | **0.410 (0.132) **** | **0.310 (0.115) **** | 0.031 (0.123) |
| **Substance addiction** | -0.420 (0.230) | -0.228 (0.200) | **-0.559 (0.218) *** |
| **Sex ^c^** | **1.346 (0.161) ***** | **1.496 (0.139) ***** | 0.055 (0.152) |
| * p<0.05; ** p<0.01; *** p<0.001; L/A = Leptin/adiponectin; MDD = Major depressive disorder; SE = Standard error; Included: ^a^ n = 172; ^b^ n = 156; ^c^ Reference = male. | | | |
| **Table S3.** Generalized linear models for the L/A ratio, leptin and IL-6 in the female study population (N=168) | | | |
|  | **L/A ratio ^a^** | **Leptin ^a^** | **IL-6 ^b^** |
| Factors | B (SE) | B (SE) | B (SE) |
| **Neuroticism score** | **0.030 (0.009) **** | **0.028 (0.007) ***** | **0.026 (0.008) ***** |
| **MDD** | -0.168 (0.159) | -0.057 (0.134) | -0.239 (0.143) |
| **Any anxiety disorder** | -0.138 (0.169) | -0.173 (0.138) | -0.164 (0.151) |
| **Antidepressants** | **0.383 (0.148) *** | 0.236 (0.123) | 0.046 (0.129) |
| **Substance addiction** | -0.333 (0.245) | -0.137 (0.207) | **-0.455 (0.221) *** |
| * p<0.05; ** p<0.01; *** p<0.001; L/A = Leptin/adiponectin; MDD = Major depressive disorder; SE = Standard error; Included: ^a^ n = 168; ^b^ n = 159. | | | |

| **Table S4.**  Spearman’s correlation coefficients for the neuroticism score and the neuroticism score subscales in the total study population (T) and the patient population (P) | | | | | | | | | | | | |
| --- | --- | --- | --- | --- | --- | --- | --- | --- | --- | --- | --- | --- |
|  | **L/A ratio** | | **Leptin** | | **Adiponectin** | | **CRP** | | **IL-6** | | **TNF-α** | |
|  | T | P | T | P | T | P | T | P | T | P | T | P |
| **Neuroticism score** | **0.19 **** | **0.18 *** | **0.19 **** | **0.19 *** | -0.05 | -0.06 | 0.11 | 0.06 | **0.18 *** | 0.11 | -0.05 | 0.03 |
| **Psychic trait anxiety** | 0.06 | 0.05 | 0.04 | 0.05 | -0.04 | -0.02 | 0.07 | 0.02 | 0.09 | <0.01 | -0.04 | 0.02 |
| **Somatic trait anxiety** | 0.12 | 0.06 | 0.12 | 0.07 | 0.02 | 0.03 | 0.13 | 0.09 | 0.10 | 0.03 | -0.02 | 0.01 |
| **Stress susceptibility** | **0.21 **** | **0.22 **** | **0.17 *** | **0.18 *** | **-0.15 *** | **-0.18 *** | 0.12 | 0.07 | **0.16 *** | 0.12 | -0.06 | 0.03 |
| **Mistrust** | **0.17 *** | **0.16 *** | **0.18 **** | **0.16 *** | -0.03 | -0.10 | 0.06 | 0.01 | 0.13 | 0.08 | -0.08 | -0.03 |
| **Embitterment** | **0.19 **** | **0.20 *** | **0.20 **** | **0.21 **** | -0.02 | -0.01 | **0.15*** | 0.09 | **0.19 **** | 0.12 | -0.05 | <-0.01 |
| **Lack of assertiveness** | 0.05 | 0.04 | 0.06 | 0.07 | 0.04 | 0.07 | <0.01 | 0.02 | 0.14 | 0.14 | 0.05 | 0.10 |
| Spearman’s correlation coefficients were used to determine statistical significance. * p<0.05; ** p<0.01; T = Total study population, including both patients and healthy controls; P = Patient population; L/A = Leptin/adiponectin; SSP = Swedish universities Scales of Personality | | | | | | | | | | | | |

| **Table S5.**  Spearman’s correlation coefficients for the neuroticism score and the neuroticism score subscales in the female study population (F) and the male study population (M) | | | | | | | | | | | | |
| --- | --- | --- | --- | --- | --- | --- | --- | --- | --- | --- | --- | --- |
|  | **L/A ratio** | | **Leptin** | | **Adiponectin** | | **CRP** | | **IL-6** | | **TNF-α** | |
|  | F | M | F | M | F | M | F | M | F | M | F | M |
| **Neuroticism score** | **0.26 **** | 0.15 | **0.27 **** | 0.14 | -0.04 | <0.01 | 0.13 | 0.12 | **0.20 *** | 0.12 | -0.09 | 0.05 |
| **Psychic trait anxiety** | **0.20 *** | 0.18 | **0.23 **** | 0.20 | 0.03 | <-0.01 | 0.11 | 0.15 | 0.13 | 0.09 | -0.10 | 0.11 |
| **Somatic trait anxiety** | **0.20 **** | -0.01 | **0.20 **** | 0.08 | -0.05 | 0.27 | **0.17 *** | 0.07 | **0.16 *** | -0.04 | -0.01 | -0.08 |
| **Stress susceptibility** | **0.25 **** | 0.24 | **0.21 **** | 0.23 | **-0.16 *** | -0.11 | 0.11 | 0.24 | **0.18 *** | 0.13 | -0.05 | -0.10 |
| **Mistrust** | **0.24 **** | -0.07 | **0.23 **** | -0.15 | -0.07 | 0.05 | 0.12 | -0.15 | 0.15 | 0.11 | -0.13 | 0.18 |
| **Embitterment** | **0.20 **** | 0.16 | **0.22 **** | 0.12 | -0.01 | -0.08 | 0.15 | 0.10 | **0.19 *** | 0.17 | -0.10 | 0.17 |
| **Lack of assertiveness** | 0.04 | 0.24 | 0.11 | 0.22 | 0.13 | -0.15 | -0.03 | 0.20 | 0.14 | 0.16 | 0.01 | 0.19 |
| Spearman’s correlation coefficients were used to determine statistical significance. * p<0.05; ** p<0.01; F = Female study population, including both patients and healthy controls; M = Male study population, including both patients and healthy controls; L/A = Leptin/adiponectin; SSP = Swedish universities Scales of Personality | | | | | | | | | | | | |

**Figure S1.** Flow-chart of the selection process

Controls available from the UPP (n=60)

Patients available from the UPP (n=228)

Male study population (n=50)

Female study population (n=168)

Fulfilled ≥1 exclusion criteria (n=14)

- Coeliac disorder (n=1)
- Current psychiatric disorder (n=8)
- Psychotropic medication (n=3)
- Incomplete neuroticism score (n=2)

Fulfilled ≥1 exclusion criteria (n=55)

- Systemic Inflammatory Disorder (n=9)
- Diabetes Mellitus (n=3)
- Pregnancy (n=1)
- Testosterone treatment (n=1)
- > 4 months between the health examination and blood sample collection (n=1)
- Coeliac disorder (n=7)
- Incomplete diagnostic assessments (n=15)
- Incomplete neuroticism score (n=4)
- Bulimia nervosa (n=16)
- Anorexia nervosa (n=3)

A

Excluded because of high HbA1c (n=1)

Patient population (n=172)

Control population (n=46)

Total study population (n=218)
